# Toll-Like Receptors: Role in Dermatological Disease

**DOI:** 10.1155/2010/437246

**Published:** 2010-08-22

**Authors:** Aswin Hari, Tracy L. Flach, Yan Shi, P. Régine Mydlarski

**Affiliations:** ^1^Immunology Research Group, University of Calgary, Calgary, AB, Canada T2N 4N1; ^2^Department of Microbiology & Infectious Diseases, University of Calgary, Calgary, AB, Canada T2N 4N1; ^3^Department of Medicine, University of Calgary, Calgary, AB, Canada T2N 4N1

## Abstract

Toll-like receptors (TLRs) are a class of conserved receptors that recognize pathogen-associated molecular patterns (PAMPs) present in microbes. In humans, at least ten TLRs have been identified, and their recognition targets range from bacterial endotoxins to lipopeptides, DNA, dsRNA, ssRNA, fungal products, and several host factors. Of dermatological interest, these receptors are expressed on several skin cells including keratinocytes, melanocytes, and Langerhans cells. TLRs are essential in identifying microbial products and are known to link the innate and adaptive immune systems. Over the years, there have been significant advances in our understanding of TLRs in skin inflammation, cutaneous malignancies, and defence mechanisms. In this paper, we will describe the association between TLRs and various skin pathologies and discuss proposed TLR therapeutics.

## 1. Introduction

Toll receptors were first discovered in *Drosophila* where they are involved in embryogenesis [1]. They were later shown to assist in innate immunity where their activation resulted in the production of antimicrobial peptides. Thus, structurally similar receptors found in other species were named toll-like receptors (TLRs) [1, 2]. In mammals, TLRs represent a family of pattern recognition receptors (PRRs) that recognize distinct, conserved microbial components and permit cells to recognize self from nonself in immune activation [1, 3]. The TLR family represents a known group of at least 10 human transmembrane proteins which are essential for innate immunity [4]. Immune cells such as monocytes, macrophages, dendritic cells, granulocytes, and nonimmune cells like keratinocytes express TLRs. Predictably, TLRs are mostly found on cells that initiate the primary immune response. TLRs detect a variety of PAMPs which include Lipopolysaccharide (TLR 4), double-stranded (DS) RNA (TLR 3), and single-stranded (SS) RNA (TLR 7) [5, 6]. These receptors are located on the cell surface, the endocytic vesicle membrane, or intracellular organelles [6]. TLRs have an ectodomain composed of leucine-rich repeats (LRRs) that may bind directly to ligands [2]. Alternately, accessory molecules may also be involved in ligand binding in addition to a cytoplasmic toll/interleukin-1 (IL-1) receptor (TIR) domain, which interacts with TIR-domain-containing adaptor molecules [6]. In the case of human TLR 4, two accessory molecules CD14 and MD2 are essential for LPS recognition [2, 7, 8]. TLRs have the ability to initiate a rapid and potent response upon ligand engagement. While TLRs are single-membrane spanning noncatalytic receptors; different TLRs are able to pair up with each other to expand their range of recognition targets. For instance, TLR 1 and 2 pair up and sense peptidoglycans. While most TLRs signal through the Myd88 pathway (Myeloid differentiation factor-88), TLR 3 and 4 utilize the TRIF pathway (TIR domain-containing adapter protein that induces IFN-*β*), and TRAF 6 (TNF receptor-associated factor 6) was found to be the additional transducer for TLR 7 and 9 [9, 10]. Ensuing these signalling intermediates, TLRs eventually trigger nuclear factor kappa-light-chain-enhancer of activated B cells-(NF-*κ*B) dependent and interferon regulatory factor (IRF-) dependent activation events [9]. Activation of these transcription factors results in induction of immune and inflammatory genes, namely, tumor necrosis factor alpha (TNF-*α*) and type I interferons (IFNs) [9]. At these events, TLR-mediated activation is quite similar to that of another important inflammatory cytokine receptor, interleukin-1 receptor (IL-1R). TLRs and IL-1R share the Myd88 adaptor molecule and promote the production of proinflammatory cytokines such as leukotrienes (LTs), prostaglandins, and chemokines [4]. Once activated by TLRs, immune cells initiate phagocytosis/killing of pathogens, cytokine, and chemokine production, leukocyte activation, and antigen presentation to T cells, thereby, initiating an adaptive immune response. An in-depth insight into different TLRs and their associated characteristics such as ligands, cytokines induction profile and functions have been previously explained by others [11]. TLRs can modulate adaptive response with respect to Th1 and Th2 as reported in study, by Medzhitov et al. [2, 12]. The study found that the mice lacking Myd88 were defective in giving rise to antigen-specific Th1 T helper cells but could initiate a normal Th2 response. All TLR ligands trigger Th1 response which seem essential in defence against microbial antigens that are viral, bacterial, and fungal in nature, in contrast to specific interaction against antigens such as helminths that require a Th2 response [2].

**Figure 1:**
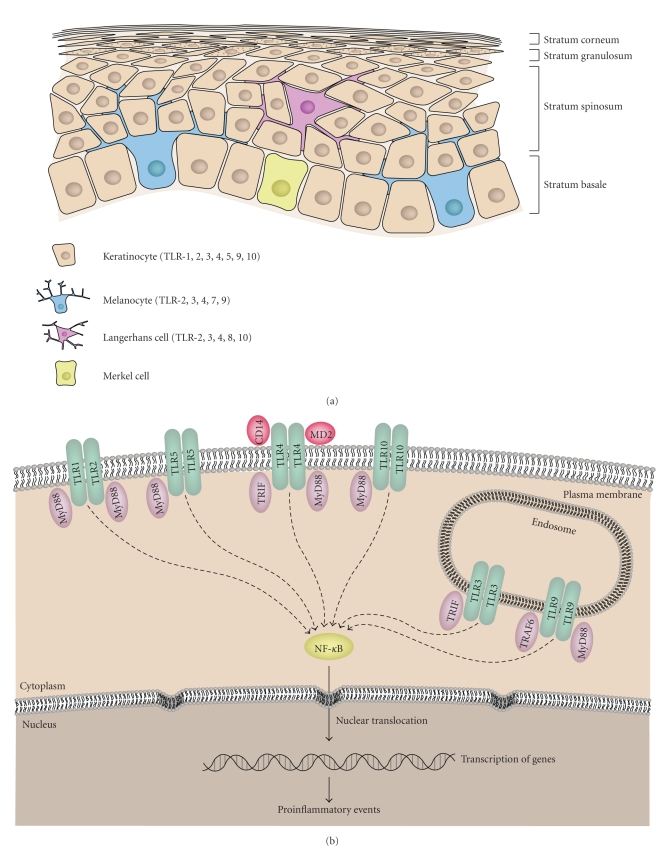

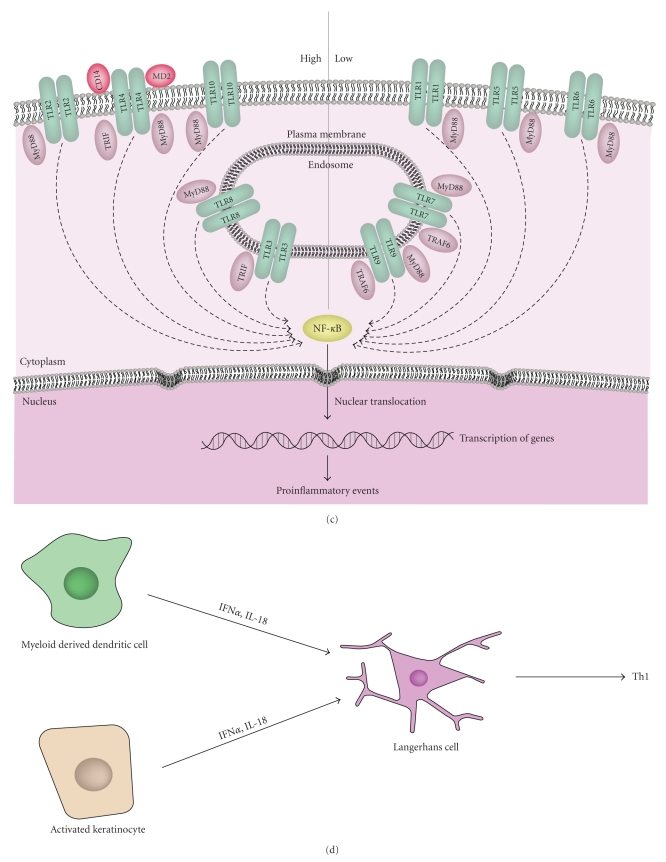

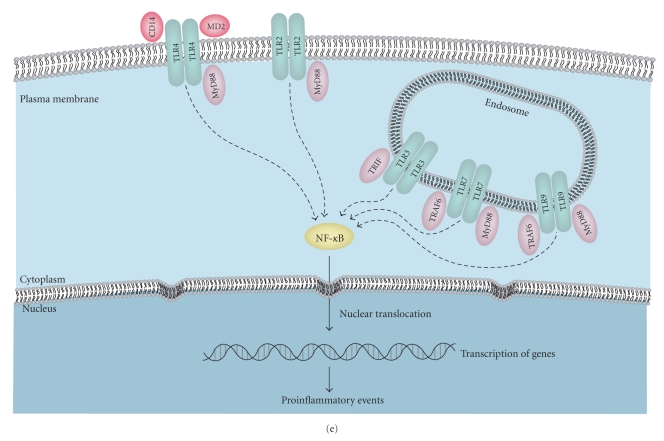
(a) Cross-section of epidermis. Keratinocytes are present throughout the skin; other epidermal cell types include Langerhans cells, melanocytes, and merkel cells. (b) Keratinocytes express TLRs 1, 2, 3, 4, 5, 9, and 10. MD2: TLR4-associated molecule; MyD88: myeloid differentiation factor 88; NF-*κ*B: nuclear factor-*κ*B; TRAF-6: TNF receptor-associated factor-6; TRIF: TIR domain-containing adapter protein that induces IFN-*β*. (c) Langerhans cells express high levels of TLRs 2, 3, 4, 8, and 10. but express low levels of TLRs 1, 5, 6, 7, and 9. (d) Upon activation, myeloid-derived dendritic cells and to a lesser extent keratinocytes release IFN*α* and IL-18 which stimulate Langerhans cell maturation. With antigen stimulation the Langerhans cell induces a Th1 response. (e) In response to TLRs 2, 3, 4, 7, and 9 melanocytes initiate proinflammatory events via the illustrated pathways.

The skin is the first line of defence against a variety of physical and biological assaults. It shields the body from harmful chemicals, physical trauma, and ultraviolet (UV) radiation. In addition, it is essential in maintaining temperature and homeostasis and in our sensing of the environment. The skin has evolved into a complicated, yet tightly regulated system appropriate for the complex functionalities associated within it. The outermost layer of the skin is the epidermis and is composed of four main types of cells: keratinocytes, Langerhans cells, melanocytes, and Merkel cells (Figure 1(a)). Keratinocytes are capable of proliferating and maintaining the outer layer which is composed of a stratified epithelial zone and a water resistant layer of lipids on the outmost surface. The melanocytes residing in the skin provide the melanin pigment that is essential for protection against UV radiation. Interspersed between keratinocytes and melanocytes are the Langerhans cells (which function as cutaneous antigen presenting cells (APCs)) and intraepithelial T lymphocytes. Merkel cells are oval sensory cells found in the skin. They play a role in light touch discrimination and may have a neuroendocrine function. The epidermis is anchored to the dermis below via connective tissues. The dermis is rich in blood vessels, nerves and has abundant fibroblasts, dendritic cells, macrophages, and lymphocytes. 

 The repertoire of TLRs found on each of these three epidermal cell types varies (Figure 1(a)). Even though the precise role against pathogens is poorly understood, in general, TLRs in the skin respond to their ligands by activating NF-*κ*B and producing cytokines [13]. Keratinocytes are reported to express TLRs 1, 2, 3, 5, 9, and 10 [14–21]; the evidence supporting TLR 4 expression in keratinocytes is conflicting [14, 18–20] (Figure 1(b)). As an example of TLR 2 engagement, human keratinocytes mainly signal through TLR 2 once activated with *Staphylococcus aureus *(*S. aureus*) or *Candida albicans * [15, 20]. Keratinocytes, treated with *S. aureus, *transcribe NF-*κ*B controlled genes (cyclooxygenase-2, nitric oxide synthetase, and IL-8) and produce enhanced levels of IL-8, nitric oxide, and chemokines [15]. Ligands for TLR 2, 3, and 5 stimulate production of matrix metalloproteases (MMPs) 1 and 9, along with activation of the NF-*κ*B pathway [22]. The expression of these cytokines is needed for proper recruitment, inflammation, and damaged tissue remodelling [22]. Translocation of NF-*κ*B and the associated events permits keratinocytes to stimulate dendritic cell maturation and enhance antigen presentation [23]. Activated keratinocytes are also important epidermal cytokine producers which mobilize leukocytes, signal other cutaneous cells and attract neutrophil granulocytes and professional killer cells [20]. Langerhans cells express significant levels of TLRs 2, 3, 4, 8, and 10 and low levels of TLRs 1, 5, 6, 7, and 9 [24] (Figure 1(c)). DS RNA, present in some viruses, induces a particularly robust response in Langerhans cells, implicating its role in anti-viral immunity via TLR 3 [25]. Additionally, Langerhans cells can be activated indirectly by activated myeloid derived dendritic cells and keratinocytes (Figure 1(d)). For example, they were found to mature and initiate a Th1 response in the presence of keratinocytes that secreted IFN*α* and IL-18 upon exposure to antigen, such as DS RNA or polyinosinic-polycytidylic acid (poly IC) stimulation [23]. Lastly, human melanocytes have been shown to express TLRs 2, 3, 4, 7, 9 (at the protein level), and respond to TLR 4 ligands by MMP induction [25, 26] (Figure 1(e)). When stimulated by TLR ligands, human melanocytes can: (1) release IL-6 and IL-8 cytokines, (2) enhance chemokine (CCL2, CCL3, and CCL5) mRNA production, (3) upregulate phosphorylated I*κ*B*α* (nuclear factor of kappa light polypeptide gene enhancer in B-cells inhibitor, alpha), and (4) promote translocation of NF-*κ*Bp65 to the nucleus [25].

TLRs are increasingly being implicated in many immune and inflammatory diseases, cancer, and wound healing. For example, TLR 2, TLR 4, TLR 5, and TLR 7 are implicated in tumor metastasis, sepsis, radioprotection, and systemic lupus erythematosus, respectively [83]. Wound healing was found to be affected in Myd88^−/−^ mice suggesting a synergistic role for TLRs in this process [84]. That study revealed interplay between purinogenic receptor signalling and Myd88^−/−^ pathways but did not explore IL-1 cytokine profile. The involvement of Myd88 hints at an important function of associated TLRs, especially TLR 2 and 4, in slain tissue remodelling. Lai et al. revealed a novel mechanism by which bacterial products modulate local inflammation in a TLR 2-dependent manner [85]. Understanding TLR signalling pathways, their structural interaction with ligands, and inhibition strategies may provide avenues for potential clinical intervention [83]. In order to clinically manipulate TLRs, pharmacologics should alter TLR activity through receptor antagonists, receptor agonists and signal transduction inhibitors. Moreover, neutralizing antibodies, monotherapies and adjuvancy may also be used to target TLRs. E5564 (Eritoran) is a good example of a current pharmacological (TLR 4 antagonist) agent which is being clinically tested for treatment of Gram-negative endotoxemia and sepsis [83]. TLR-mediated activation or dysfunction has been attributed to exacerbation of different diseases. The manipulation of specific TLRs may therefore lead to the development of novel therapies for autoimmunity, cancer, and inflammatory disease. Next we will discuss how TLRs have been linked to dermatological disease and their proposed therapeutic role.

## 2. TLRs in Dermatologic Disease

### 2.1. Atopic Dermatitis and Allergic Contact Dermatitis

Atopic dermatitis (AD), which affects up to 20% of the pediatric population, is a chronic inflammatory skin disease characterized by pruritus, eczematous lesions, xerosis, and lichenification. It often forms part of the atopic triad composed of allergic rhinitis, asthma, and eczema [86, 87]. Patients suffering from AD have greater susceptibility to bacterial, viral, and fungal infections; in fact, *S. aureus *has been associated with its flares and severity [71, 88, 89]. In addition to *S. aureus*, the best characterized infectious agent of AD, strains of *Candida* species have a strong ability to colonize atopic skin, and viruses such as herpes simplex virus can aggravate the infection and exacerbate the disease [90]. These pathogens express microbial products that stimulate TLR 1, 2, 6, and 9. Recent studies show a strong association between TLR 2 and the symptoms of severe AD in some populations [29, 30]. The presence of a single nucleotide polymorphism (SNP) R753Q in the TLR 2 allele has been reported to be associated in patients with severe AD and whose skin was prone to *S. aureus* infection [11, 91]. The role of R753Q in AD has been confirmed in the cytokine-based profiling of patients where stimulation of TLR 2 induced altered production of IL-6 and IL-12 [92]. Conversely, a study in a German population found no significant effect of TLR 2 and 4 polymorphisms in relation to susceptibility for AD [93]. On the other hand, polymorphism C-1237T in the TLR 9 gene has been attributed as the cause for impairment of immunity in some cases of AD [31]. Taken together, these studies suggest that there is a defect in TLR 2/9. These defects may be genetic (dysfunctional proteins) or functional (attenuation of regulatory pathways) [31, 32, 87]. This defective recognition of pathogenic antigens by TLRs renders greater susceptibility of AD lesions to various bacterial and viral infections [1]. However, there has been no significant correlation between TLRs 1 and 6 (recognize peptidoglycans, and lipoproteins resp.) and AD [30, 94, 95]. Although correlation between TLRs 2, 9 and AD has been reported, another study using monocyte-derived DCs found no relation between polymorphism, function of TLR 2-4 with respect to AD [96]. Allergic contact dermatitis (ACD), another eczematous process, is a type IV delayed hypersensitivity reaction seen in the skin typically after sensitization by haptens [97]. The bacterial infections and haptens binding with self-proteins in the skin leads to activation of DCs stimulating a specific autoreactive CD8 response against the epidermis in a TLRs 2-, 4- and 9-dependent fashion [32, 98, 99].

 In the case of AD, defective epithelial barrier function, receptor expression, and signalling pathways (altered cytokine production) play a role in disease progression [94, 100–102]. AD and ACD are routinely treated with emollients and topical steroids (for skin flares). Topical calcineurin inhibitors (TCIs), such as pimecrolimus and tacrolimus, are used to treat AD by modulating T cell activation [102–104]. Pimecrolimus is capable of enhancing the ability of keratinocytes to inhibit pathogen growth. Although pimecrolimus and tacrolimus, have been shown to effectively reduce the symptoms observed compared to placebos, the long-term effects need to be studied in more detail before declaring the treatments safe [102, 103]. Unfortunately TCIs received a black box warning from the FDA for possible links to carcinogenicity. 

 Another feasible option for the treatment of severe cases of AD is phototherapy. In patients with acute flares, ultraviolet A (UVA), ultraviolet B (UVB), or a combination of UVA and UVB therapy may be administered, often with concomitant topical corticosteroids, with a reasonable amount of success [102]. Phototherapy with narrow and broad band UV radiation is noted to be effective in treating AD as radiation interferes with the immune function and inflammatory process of the local milieu [105–107]. This is relevant to TLRs in the light of findings that TLR 2- and 4 mediated production of cytokines plays a role in immunosuppression along with T regulatory cells [50].

#### 2.1.1. Psoriasis

Psoriasis is a common, chronic, relapsing, inflammatory skin disorder with a strong genetic basis. Chronic plaque psoriasis is the most common form of the disease, but other manifestations include guttate, inverse, pustular, erythrodermic, and psoriatic arthritis. Psoriasis is characterized by epidermal hyperproliferation (acanthosis), marked hyperkeratosis with parakeratosis (abnormal maturation), loss of the granular layer, vascular dilatation, and inflammatory cell infiltration. Abnormalities in biochemical, inflammatory, and immunological markers are commonly identified. In the skin of psoriasis patients, there is increased antigen presentation, T cell proliferation, Th1 and Th17 cytokine production, and angiogenesis. Inappropriate recognition of self-nucleic acids in addition to type I IFNs (IFN-*α*/*β*) production by plasmacytoid DCs (pDCs) can lead to psoriasis [108]. pDCs are an important cell population in this condition, because they are 16% of the total dermal infiltrate in psoriatic skin lesions whereas they are rare in atopic dermatitis and nonexistent in normal skin [108]. As an example, mechanical injury towards prepsoriatic skin promotes the release of cationic antimicrobial peptide (LL_37 _) by keratinocytes [61]. The overexpressed LL_37 _ binds to extracellular self-DNA and forms aggregates that enter pDCs, activate TLR 9, and trigger type I IFN production. Type I IFNs produced by pDCs support myeloid dendritic cell maturation and eventual autoreactive T cell activation leading to psoriatic skin lesions, in a self-sustaining feedback loop. Concurrently, Th17 cells produce IL-22 and IL-17 which stimulate epidermal hyperplasia and neutrophil recruitment and further enhance inflammation. IFN-*α* (type 1 IFN) in addition to ssRNA (a simulator of viral infection) also acts as an important trigger for DC activation in psoriasis [109]. Another important growth factor expressed during psoriasis is TGF-*α*, which upregulates TLRs 5 and 9 expression and function in human keratinocytes [110]. Heat shock proteins (i.e., HSP60) are suspected immunogenic proteins that are heavily expressed by epidermal keratinocytes of guttate and plaque psoriasis in comparison to normal skin [111]. HSP60 may subsequently trigger TLR 2 and TLR 4 to stimulate innate and adaptive immunity which develops and/or aggravates psoriasis. The A domain of fibronectin (keratinocyte derived) may act on the TLR 4 pathway in APCs (Langerhans cells) to cause maturation, TNF-*α* and IL-12 secretion, and antigen presentation to autoreactive T cells [112]. Interestingly, anti-keratin 16 antibodies are highly concentrated in the serum of psoriasis patients and may be involved in chronic inflammation. Keratinocytes incubated with mouse antikeratin 16 monoclonal, antibodies have increased levels of TLR 2, TLR 4, involucrin, and NF-*κ*B nascent polypeptide-associated complex mRNA [113]. As implied earlier, psoriasis is a T cell-mediated disease caused in part by activated T cells interacting with antigen-presenting cells (APCs) in addition to IFN-*γ*, IL-1, and TNF-*α* effects [114, 115]. T cells are distinctly compartmentalized in the different skin layers in psoriatic lesions [116]. CD4^+^/Th1 T cells are, for example, located in the upper dermis unlike CD8^+^/Th1 T cells that are found in the epidermis [117]. T cells are functionally important since cyclosporine A (an immunosuppressant which binds the cyclophilins of T lymphocytes), as well as CTLAflg, anti-CD4 antibodies, DAB389IL-2, and alefacept (all T cell-based immunomodulators) are highly effective therapies for psoriasis [118–122]. Further support is provided in rare instances of psoriasis transfer to recipients of bone marrow transplants from donors with the disease and similar episodes in xenotransplantation models [123, 124].

In regard to TLR expression, keratinocytes in the epidermis constitutively express TLR 1, 2, and 5. In psoriasis lesions, the expression of TLR 1 and TLR 2 on keratinocytes is further upregulated [62]. Keratinocytes in human psoriatic skin, activated by TLR 2, 3, and 4 ligands exhibited NF-*κ*B nuclear translocation and release of TNF-*α* and IL-8 [63]. An immunohistochemistry-based profile of psoriatic skin also demonstrates the overexpression of TLR 2 in epidermal and dermal DCs and the enhanced TLR 2 expression in basal layer keratinocytes. It enhanced TLR 4 expression in epidermal and dermal DCs, in addition to accumulation of CD14-positive macrophages [125]. TLRs 7 and 8 signalling have also been implicated in psoriatic exacerbations [112] since imiquimod (a TLR 7/8 agonist) aggravates pathological symptoms. 

Topical immunosuppressive therapies such as topical corticosteroids (i.e., budesonide), pimecrolimus, and tacrolimus are currently used for treating inflammatory skin diseases like atopic dermatitis. They function by suppressing IL-8 and TNF-*α* mRNA expression and either induce (budesonide) or suppress (tacrolimus) TLR 2 mRNA expression in human keratinocytes [126, 127]. Although further studies are required, it appears that the clinical use of systemic and topical retinoids helps to control psoriatic inflammation through TLR 2 inhibition [50]. Monomethyl fumarate (fumaric acid ester) is also used as an immunotherapy for psoriasis; markedly, it interferes with LPS signalling through TLR 4 in dendritic cells, inhibits NF-*κ*B activation, decreases IL-12p70 and IL-10 production, and modulates monocytes-derived DC polarization [128]. Furthermore, the TLR 7/8 agonist imiquimod, which was considered a potential therapy, resulted in exacerbated psoriatic lesions, increased DCs infiltration, and elevated IFN-*γ*. Another example of potential treatment for psoriasis is the use of anti-inflammatory pharmacologics such as salicylates (as well as other nonsteroidal anti-inflammatory drugs) and parthenolide, which block IkappaB kinase (IKK-1 and IKK-2) activity [123]. IKK-1 and IKK-2 are part of the TLR signalling cascade [129]. Although advances are being made and compounds are being clinically tested to target the common signaling pathways of the TLR superfamily to prevent p38 mitogen-activated protein, NF-*κ*B activity, and TNF production, caution must be exercised when testing these TLR therapies [129].

#### 2.1.2. Acne Vulgaris

Sebaceous glands are a holocrine gland that secretes sebum [130] formed by the breakdown of glandular cells. Full blown acne manifests in the pilosebaceous follicle and is attributed to increased sebum excretion, sebum lipid alteration, androgen and neuropeptide activities, follicular hyperkeratinization, dsyregulation of cutaneous steroidogenesis, and growth of anaerobic, gram positive *Propionibacterium acne* (*P. acnes)* [131, 132]. *P. acnes* helps sustain inflammation by TLR-mediated induction of cytokines, upregulation of adhesion molecules, and chemokine mediated recruitment of immune cells [133]. Immune cells such as TLR 2 expressing macrophages (perifollicular and peribulbar) tend to surround pilosebaceous follicles in acne lesions; subsequent TLR 2 triggering due to *P. acnes* results in IL-6, IL-8, and IL-12 cytokine production [4]. The pro-inflammatory response in acne lesions seems to be largely initiated by TLR 2 since the lipid components from the cell wall of *P. acnes *also trigger TLR 2 activation in monocytes [4]; almost all of the TLR 2-expressing cells in acne lesions are derived from CD14 monocytes, suggesting active monocyte recruitment. Monocytes are, in turn, stimulated to produce TNF-*α*, IL-12, IL 1-*β*, and IL-8 [4, 133], which are all major pro-inflammatory cytokines/chemoattractants. Consequently, neutrophils and lymphocytes are attracted to the follicle and exaggerate the observed inflammation. Acne inflammation may also develop because *P. acnes* stimulates the expression of TLR 2, 4 and CD14 in human keratinocytes [27]. Keratinocytes and sebocytes, located near the pilosebaceous unit, may be capable of detecting either pathogens or abnormal lipids [27]. Human SZ95 sebocytes, in particular, express innate immune molecules such as TLR 2, TLR 4, IL-1*β*, IL-6, IL-8 [27], CD1d, and CD14 [131]. As a result of TLR stimulation due to *P. acnes*, pro-inflammatory cytokines, chemokines, antimicrobial lipids [132] and antimicrobial peptides (i.e., cathelicidin, defensin-1, defensin-2, and psoriasin) [134–137], and human B-defensin-2 are produced by these cells [138]. 

In acne vulgaris, TLR 2 activation can induce robust inflammation as well as cellular apoptosis and tissue injury [4, 28]. Pharmacological agents have been designed to target inflammation through downregulation of TLR expression and function. Retinoids (a class of vitamin A-derived compounds) bind retinoic acid receptors (RAR) and modulate keratinocyte maturation [139–141]. When primary human monocytes are treated with ATRA (all-trans retinoic acid), TLR 2 and CD14 are downregulated without any change in expression of TLR 1 and 4 [142]. Further, ATRA pre- and cotreatment of monocytes inhibited the ability of TLR 1/2 ligands to trigger cytokine production [142]. In response to *P. acnes*, ATRA-treated monocytes result in cytokine downregulation of IL-12p40, TNF-*α*, and IL-6 [142]. Other antiacneic drugs such as retinoic acid, ZNSO4, doxycycline, nicotinamide, nitroimidazole, retinol, and isotretinoin prevent O_2_^•^ production, IL-8 release and keratinocyte apoptosis [143]. Nicotinamide, through interaction with TLR 2 on primary keratinocytes and immortalized HaCaT cells, significantly depresses IL-8 production (both at mRNA and protein levels) in a dose-dependent manner [144]. It appears that nicotinamide's downregulation of IL-8 is accomplished at the level of transcription by reducing promoter activity. It also inhibits I*κ*B degradation (reduced NF*κ*B) and phosphorylation of Jun N-terminal (JNK) MAP kinases and extracellular receptor kinases (ERK) [144]. Current acne therapies exert their effects, at least in part, via TLRs, and newly developing TLR therapies show great potential.

### 2.2. Nonmelanoma Skin Cancers

Non-melanoma skin cancer (NMSC) is the most common form of cancer worldwide. The two major types of NMSC are basal cell carcinoma (BCC) and squamous cell carcinoma (SCC). Risk factors for the development of NMSC include exposure to high doses of UV and ionizing radiation, chemical carcinogens, immunosuppression, oncogenic viral strains, and genetic predisposition. 

 BCCs, typically occur in areas of chronic sun exposure are slow growing and rarely metastatize [145–147]. Current therapies consist of surgical removal, radiotherapy, photodynamic therapy, cryotherapy, and intralesional injections of IFN-*α* [148]. Imiquimod has been administered successfully as a topical immune modulator which induces production of IFN-*α* and IL-2, leading to a heightened immune response [33, 145, 146]. The effect is therefore expected to be mediated by TLR 7/8. Topical application of 5% imiquimod has demonstrated efficacy in several clinical trials [33–35, 145, 149–151]. Stary et al. reported observations where topical imiquimod treatment leads to induction of a specific subset of DCs which mediated destruction of BCC lesions [36, 37]. CpG ODNs are composed of repeating unmethylated CG regions causing them to resemble bacterial DNA. PF-3512676 (a synthetic CpG ODN) is also considered safe and effective in treating BCC [38], which presumably targets TLR 9. 

 The prevalence of SCC is second only to that of BCC. It is found more commonly in elderly Caucasian men with significant cumulative UV damage [147, 152–154]. Unlike BCCs, SCCs have higher rates of metastasis and tend to be more aggressive in immunosuppressed patients [152]. SCCs are typically treated by surgery, radiation therapy and other chemotherapeutic modalities [154]. Combination therapy consisting of immunosuppressive agents (methotrexate, bleomycin supplemented with 5-bromouracil) did not yield tangible results [155]. A similar method using interferon instead of bromouracil was reported to be much more effective [154, 156]. As in the case of many skin diseases, imiquimod has been tested for SCC mitigation. TLR 7 and 8 have been manipulated to activate the pro-inflammatory machinery against SCC [154]. The modulation of IL-1, IL-6, IL-8, and IL-12 along with promotion of a Th1 response promotes antitumor and antiviral behaviour [154]. Several studies have shown imiquimod to be a viable option to test for efficacy in treatment of SCC [68–70].

### 2.3. Melanoma

Melanoma is a malignant tumor of melanocytes which causes the majority of skin cancer-related deaths worldwide [58, 157, 158]. Exposure to UV radiation is the main risk factor for the disease [58, 157, 159]. Melanocytes have the ability to help tumor progression in melanoma by responding to hyaluronic acid fragments through TLR 4 by inducing MMP and cytokine production [59]. Although melanocytes have been shown to express other TLRs [25], TLR 9 has been targeted for modulating immune response. A study on a murine lymphoma model which revealed the potential of CpG ODNs in enhancing immunogenicity of the tumours resulted in exploitation of TLR 9 ligands for melanoma [60, 160].

Many TLR ligands are considered good adjuvant candidates as they can activate dendritic cells. Different TLR 9 agonists are now included in vaccine formulations for human trials. In earlier studies, a vaccine with antigenic peptide and IFA (incomplete Freund's adjuvant) was reported to induce a weak T cell response. On the contrary, studies with CpG ODNs produced a strong immune response against the Hepatitis B virus [161]. In clinical studies using CpG 7909 (a variant of CpG ODNs) in combination with a melanoma peptide, the vaccine resulted in 10-fold greater specific CD8 T cells compared to peptide alone [60, 161, 162]. Recently a variant of CpG 7909 named PF-3512676 was used [163] in a phase II clinical trial which yielded promising results and had a moderate safety profile but displayed some very rare adverse events. In all, TLR 9 targeting appears to be a viable option in melanoma treatment [164]. In addition, imiquimod (a TLR 7 agonist) was used to harness the potential TLR 7-based treatment for melanoma. Imiquimod is effective in both activating dendritic cells and producing tumor-specific cytotoxic T cells that arrest disease progression [165].

### 2.4. Therapeutic Drugs

#### 2.4.1. Imidazoquinolinamines—Immune Modulators

Imidazoquinoline compounds (ICs) have similar structure to nucleosides found in all living organisms. They were initially produced and tested as antiallergic drugs in rats, and were reported to be reasonably effective [166]. After extensive studies, the potential of ICs to stimulate secretion of pro-inflammatory cytokines was finally discovered [167, 168]. Toll like receptor 7/8 directs a cascade of events which leads to the release of specific cytokines [169]. Imiquimod and resiquimod are two compounds that are efficient at activating the immune system [170]. Both compounds induce secretion of IFN-*α*, TNF-*α*, IL-6, and other pro-inflammatory cytokines from APCs such as resident B cells, pDCs, and monocytes [170–173]. They achieve this response by activating TLR pathways and promoting nuclear transmigration of transcription factors like NF*κ*B and activator protein 1 (AP1). Imiquimod, the more extensively studied analogue, is known to enhance apoptotic pathways in cancer cells. It binds to the high affinity adenosine receptors and suppresses its ability to regulate negative feedback [168]. Resiquimod, a chemically related compound, exhibited 100-fold greater ability to stimulate apoptosis in preliminary studies [170]. Unfortunately, unlike imiquimod, resiquimod has not been studied extensively [50]. At the time of their discovery, imidazoquinolines were hypothesized to effectively treat Kaposi's sarcoma, human papillomavirus (HPV) and other infections [170]. Initially generated to be an antiviral agent, it was quickly utilized for its expanding therapeutic potential. Imiquimod was one of the first of such analogues to be authorized for treatment of genital warts caused by HPV [6, 26]. Imiquimod has also been used to activate the immune system in many diseases like actinic keratoses [50], BCC [35, 149–151], SCC [70, 154], and melanoma [58, 60, 165, 174–176]. Two other immunomodulators which function through TLR 7, loxoribine, and bropirimine have also been used [6]. Loxoribine, a guanosine ribonucleoside, enhances production of IFN and activates NK cells and B cells [50, 177, 178]. Bropirimine, an aryl pyrimidinone class of antineoplastic compounds, has a similar effect to loxoribine and is currently in clinical studies for treatment of carcinomas [50, 179].

#### 2.4.2. Calcineurin Inhibitors—Immune Suppressors

Calcineurin inhibitors are potent suppressors of the T cell mediated immune response due to their ability to inactivate the serine protease calcineurin. This prevents nuclear translocation of (NFAT nuclear factor of activated T cells), thereby inhibiting the synthesis of pro-inflammatory cytokines in T cells [104, 171]. Pimecrolimus and tacrolimus are ascomycin macrolactam derivatives that are categorised as inhibitors of calcium-dependent phosphatase. In the early stages of AD, the topical application of 1% pimecrolimus was deemed more effective than topical steroids [102, 171]. Different formulations of the drug are being tested for efficacy in psoriasis, vitiligo, and other skin diseases [127, 171, 180]. As previously mentioned, pimecrolimus has been shown to enhance the ability of keratinocytes to fight infection when coadministered with TLR 2/6 ligands [104]. Similar to pimecrolimus in structure and function, tacrolimus is utilized for identical purposes, albeit at lower concentrations (0.03% to 0.1%). Tacrolimus appears safe for administration at 0.1% for treatment of AD but with certain adverse side effects, such as burning sensation and pruritus observed in a small minority [103]. The drug is under trial for treatment of other diseases such as seborrheic and contact dermatitis, systemic lupus erythematosus, and bullous pemphigoid [171, 180]. Sirolimus or rapamycin is a macrolactam-like compound which inhibits IL-2 synthesis and cell cycle pathways [181]. Sirolimus, if injected along with cyclosporine A, prevents the progression of UV-mediated skin cancer [182]. The drug is under consideration in combination with other drugs for treatment of skin malignancies, hepatocarcinomas, lupus, psoriasis, dermatomyositis, graft rejection, and a genetic disorder, pachyonychia congenital [180, 183–188]. 

Table 1 lists a selected group of skin diseases and the different TLRs affiliated with each disease. The TLRs associated can either activate the immune system or promote disease progression as indicated.

## 3. Conclusion

Toll like receptors represent a conserved group of receptors which help the immune system to function properly. Different TLRs are associated with an array of skin diseases (Table 1). TLR agonists and antagonists have great potential for the treatment of allergic and inflammatory diseases. More research must discern the relationship between specific TLRs and the corresponding disease in order to harness the therapeutic potential of TLR ligands. Although studies have proven that TLR agonists like CpG can induce a robust immune response, the efficacy of the vaccines, optimization of dosage, long-term effects, and augmentation requires further study [162, 172].

## Figures and Tables

**Table 1:** Toll like Receptors in dermatologic disease.

Disease/infection	Toll like receptor associated	Associated function with the disease	References
Acne vulgaris	TLR 2, 4, CD14	TLR upregulation with eventual exacerbation of the disease	[4, 27, 28]
Atopic dermatitis	TLR 2, 9	TLR polymorphism leading to increased susceptibility	[29–32]
Basal cell carcinoma	TLR 7, 8, 9	Exacerbation of disease, target for therapy	[33–38]
Behçet's disease-vasculitis	TLR 4, 6	TLR 4 polymorphism leads to increased susceptibility, differential regulation of TLR 6 helps in progression of disease	[39, 40]
Borreliosis/Lyme disease	TLR 1/2 (heterodimers),4, 6	TLR upregulation with eventual exacerbation of the disease	[41, 42]
*Candida albicans*	TLR 2, 4-CD14, 6	TLR function and signalling leads to disease progression	[43–45]
Cutaneous graft versus host disease	TLR 4	Exacerbation disease in response to LPS	[46]
Herpes simplex/Varicella zoster	TLR 2, 3, 9	Select TLR 2 polymorphism associated with disease severity, TLR 3 and 9 help in viral clearance	[47–49]
Leprosy	TLR 1, 2	TLR function and signalling lead to disease progression	[21, 50–52]
*Lichen planus*	TLR 9	TLR upregulation with eventual exacerbation of the disease	[53, 54]
Lupus erythematosus	TLR 3, 7, 9	TLR 7 upregulation and TLR 3*, TLR 9 function helps to create autoreactive cells	[55–57]
Melanoma	TLR 4, 7, 9	TLR 4 exacerbates disease TLR 7,9-targetted for immune modulation	[25, 58–60]
Psoriasis	TLR 1–4, 5, 9	TLRs upregulated and help in creation of autoreactive T cells	[17, 61–63]
Sarcoidosis	TLR 2, 4	TLRs polymorphism associated with disease severity	[64–66]
Scleroderma	TLR 4	TLR function and signalling lead to disease progression	[67]
Squamous cell carcinoma	TLR 7, 8	Exacerbation of disease, being studied to target for therapy*	[68–70]
*Staph. Aureus*	TLR 2, 6	TLR 2 polymorphism associated with severity*, TLR 2/6 function and signalling lead to disease progression	[71, 72]
Stevens-Johnson syndrome/Toxic epidermal necrolysis	TLR 3	TLR polymorphism linked to disease severity	[73]
Syphilis	TLR 2, 4/5 (heterodimer)	TLRs activate the immune system	[26, 50, 74]
Vaccinia	TLR 2,3, 4	TLRs help in viral clearance but are targeted by viral products which suppress host defense	[71, 75, 76]
Verruca and Molluscum Contagiosum	TLR 3,7,9	TLR 3, 9 help in immune activation, TLR 7* possible association with disease exacerbation, proposed target for therapy	[77–80]
*Yersinia spp*—the plague	TLR 2, 4-CD14	Pathogen exploits TLR pathway to survive	[50, 81, 82]

*proposed association subject to further investigation.
